# Growth-coupled anaerobic production of isobutanol from glucose in minimal medium with *Escherichia coli*

**DOI:** 10.1186/s13068-023-02395-z

**Published:** 2023-10-03

**Authors:** Simon Boecker, Peter Schulze, Steffen Klamt

**Affiliations:** 1 Analysis and Redesign of Biological Networks, Max Planck Institute for Dynamics of Complex Technical Systems, Sandtorstr. 1, 39106 Magdeburg, Germany; 2 Physical and Chemical Foundations of Process Engineering, Max Planck Institute for Dynamics of Complex Technical Systems, Sandtorstr. 1, 39106 Magdeburg, Germany; 3 Present Address: University of Applied Sciences Berlin, Seestr. 64, 13347 Berlin, Germany

**Keywords:** Isobutanol, Redox balance, *Escherichia coli*, Fermentation, Acetate, Metabolic engineering, NADH

## Abstract

**Background:**

The microbial production of isobutanol holds promise to become a sustainable alternative to fossil-based synthesis routes for this important chemical. *Escherichia coli* has been considered as one production host, however, due to redox imbalance, growth-coupled anaerobic production of isobutanol from glucose in *E. coli* is only possible if complex media additives or small amounts of oxygen are provided. These strategies have a negative impact on product yield, productivity, reproducibility, and production costs.

**Results:**

In this study, we propose a strategy based on acetate as co-substrate for resolving the redox imbalance. We constructed the *E. coli* background strain SB001 (Δ*ldhA* Δ*frdA* Δ*pflB*) with blocked pathways from glucose to alternative fermentation products but with an enabled pathway for acetate uptake and subsequent conversion to ethanol via acetyl-CoA. This strain, if equipped with the isobutanol production plasmid pIBA4, showed robust exponential growth (*µ* = 0.05 h^−1^) under anaerobic conditions in minimal glucose medium supplemented with small amounts of acetate. In small-scale batch cultivations, the strain reached a glucose uptake rate of 4.8 mmol gDW^−1^ h^−1^, a titer of 74 mM and 89% of the theoretical maximal isobutanol/glucose yield, while secreting only small amounts of ethanol synthesized from acetate. Furthermore, we show that the strain keeps a high metabolic activity also in a pulsed fed-batch bioreactor cultivation, even if cell growth is impaired by the accumulation of isobutanol in the medium.

**Conclusions:**

This study showcases the beneficial utilization of acetate as a co-substrate and redox sink to facilitate growth-coupled production of isobutanol under anaerobic conditions. This approach holds potential for other applications with different production hosts and/or substrate–product combinations.

**Supplementary Information:**

The online version contains supplementary material available at 10.1186/s13068-023-02395-z.

## Background

Bio-based manufacturing of chemicals from renewable resources provide a promising alternative to replace conventional fossil-based production processes [1, 2]. In order to be economically viable, bio-based processes need to be further optimized in terms of the three main performance measures: titer, yield, and rate (TRY). However, due to the inherent trade-off between rate and yield, it is usually impossible to maximize these two factors simultaneously: while high biomass formation leads to a higher volumetric productivity (as it acts as the biocatalyst), it also lowers the product yield because less substrate can be converted to the actual target compound [3, 4].

One possibility to address this trade-off could be the application of two-stage processes, where biomass and product formation are separated in time [3, 5]. Often, these two-stage processes are implemented by an aerobic cultivation phase, where biomass accumulates. This first stage is then followed by an anaerobic production phase, where no growth can occur, e.g., due to nutrient limitation. The accumulation of high amounts of biomass in stage one leads to a higher overall volumetric productivity in the second phase where biomass acts as the catalyst to convert the provided substrate with a high yield (as none of the substrate is used for biomass formation) to the desired product. However, often it can be observed that the metabolic activity and thus productivity of growth-arrested cells drops dramatically in the second stage compared to exponentially growing cells [6–8]. This drop in metabolic activity could be avoided by forcing the cells to keep a high turnover, i.e., by applying the concept of enforced ATP wasting [3, 9–12].

Another approach to design two-stage processes to tackle the trade-off between yield and productivity could be to engineer cells, that are, in the second (production) phase, still able to grow while producing the desired product, but with a low biomass and high product yield [4]. Although at lower growth rates, enabling the cells to grow would help to maintain a higher metabolic activity and thus a higher overall productivity. Furthermore, during the cultivation process more and more biomass would accumulate, again leading to a higher volumetric productivity over time.

However, this second approach is not easy to implement, as it would require externally triggered dynamic changes in the metabolism during the fermentation. Furthermore, it is not possible for all combinations of production organism, product, and substrate. A prominent example is the fermentative conversion of glycerol to ethanol by *Escherichia coli* [13]. While *E. coli* is able to convert glycerol to ethanol (and formate/CO_2_ + H_2_) anaerobically, biomass formation under these conditions is not possible due to a redox imbalance. To restore cell growth, small amounts of oxygen, other external electron acceptors (e.g., nitrate, fumarate), or complex media additives need to be provided. Recently, it was shown that the addition of small amounts of acetate also resolve the redox imbalance in minimal medium and enables growth [14].

**Fig. 1:**
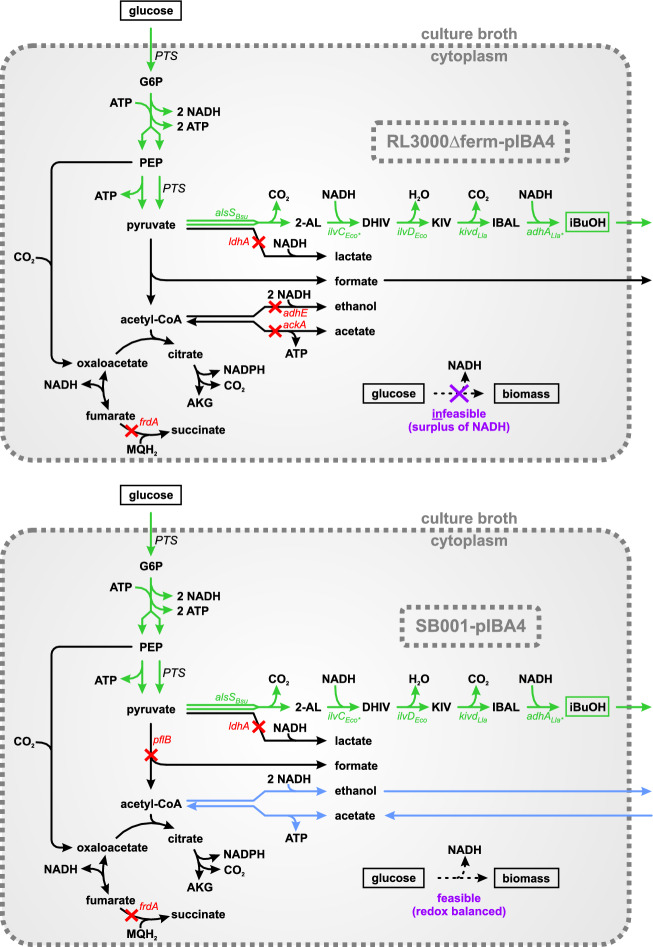
Metabolic map of the two investigated iBuOH producer strains RL3000Δferm-pIBA4 (top) and SB001-pIBA4 (bottom) for anaerobic conditions. The (green) gene names indicate the heterologous genes that were introduced via plasmid pIBA4 [*alsS*_*Bsu*_ encodes the acetolactate synthase (ALS), *ilvC *_Eco*_ encodes the ketol-acid reductoisomerase (KARI), *ilvD *_*Eco*_ encodes the dihydroxy-acid dehydratase (DHAD), *kivd*_*Lla*_ encodes the 2-ketoacid decarboxylase (KDC), and *adhA *_Lla*_ encodes the alcohol dehydrogenase (ADH)]. Red crosses and the corresponding red gene names indicate the deleted pathways. Green arrows indicate the redox-balanced pathway from glucose to iBuOH. Blue arrows indicate the pathway from acetate to ethanol, oxidizing 2 molecules of NADH and consuming 1 molecule of ATP per molecule of acetate. The purple cross indicates the redox-imbalance and thus infeasibility of cell growth in strain RL3000Δferm-pIBA4. *2-AL* 2-acetolactate, *AKG* α-ketoglutarate, *DHIV* 2,3-dihydroxyisovalerate, *G6P* glucose 6-phosphate, *IBAL* isobutyraldehyde, *iBuOH* isobutanol, *KIV* 2-ketoisovalerate, *MQH*_*2*_ menaquinol, *PEP* phosphoenolpyruvate

A similar example is the fermentative isobutanol (iBuOH) production from glucose by *E. coli* [15]. The first successful implementation of the heterologous pathway for this promising biofuel and platform chemical in *E. coli* relied on aerobic cultivation conditions due to an imbalanced cofactor utilization of the involved enzymes [16]. Two enzymes of the pathway (ketol-acid reductoisomerase (KARI) and alcohol dehydrogenase (ADH)) required NADPH as cofactors while two NADH are being formed during glycolysis (Fig. 1). This issue could be partially resolved by substituting the involved native NADPH-dependent ADH YqhD from *E. coli* with the NADH-dependent ADH AdhA from *Lactococcus lactis* [17]. However, small amounts of oxygen were still needed to resolve the redox cofactor imbalance caused by KARI. Later, the cofactor specificity of KARI was engineered and could be successfully switched from NADPH to NADH [18], through which an anaerobic synthesis of iBuOH became possible. However, the generated *E. coli* strain was cultivated in medium supplemented with large amounts of yeast extract making the overall process economically questionable and indicating that anaerobic synthesis in minimal medium was problematic. Later, the iBuOH pathway was further enhanced by increasing the activity and optimizing the expression level of the involved enzymes [18–20]. Comprehensive summaries of the performance parameters reached with different *E. coli* strains (and other organisms), substrates, media additives, and culture conditions can be found in [21, 22]. The *E. coli* strain RL3000Δferm-pIBA4 [19] exhibits one of the best performances in regard to iBuOH production so far, but reached, under anaerobic conditions in defined minimal medium, only very low biomass concentrations not relevant for industrial application. For cultivations in a bioreactor with higher biomass concentrations, yeast extract was again added to the medium [19], making a fair comparison of performance parameters with other strains or process conditions challenging [21, 23].

In our lab, strain RL3000Δferm-pIBA4 did not grow at all anaerobically in defined minimal medium. In line with Trinh and co-workers [24, 25], we hypothesized that, even though the iBuOH pathway itself is redox balanced, biomass formation leads to a redox imbalance that needs to be resolved for cell growth. As already mentioned above, recently we could show that small amounts of acetate taken up by *E. coli* and converted to ethanol via acetyl-CoA enabled for the first time anaerobic growth of this bacterium on defined minimal medium with glycerol as substrate [14]. Here, the uptake of one molecule of acetate costs one molecule of ATP but via conversion to ethanol, two molecules of NADH are consumed. To test whether providing acetate as co-substrate would also enable anaerobic growth of an *E. coli* iBuOH producer strain, we generated a new background strain: analogous to the RL3000Δferm-pIBA4 strain [19], lactate and succinate production were blocked by *ldhA* and *frdA* deletions. However, as a key difference to the RL3000Δferm-pIBA4 strain, *ackA* and *adhE* were kept active to allow uptake of acetate and its conversion to ethanol via acetyl-CoA. In addition, to prevent fermentation of glucose to formate, ethanol, and acetate, the pyruvate formate lyase gene (*pflB*) was deleted. Thus, the generated strain SB001 (*ΔpflB ΔldhA ΔfrdA*) is able to secrete ethanol, but the latter can only be synthesized from the co-substrate acetate and not from the main substrate glucose. SB001, when transformed with the iBuOH production plasmid pIBA4, indeed showed robust exponential growth (*µ* = 0.05 h^−1^) in minimal medium supplemented with acetate while no growth could be observed in acetate-free medium. Glucose was consumed at a relatively high rate (*r*_Glc_ = 4.8 mmol gDW^−1^ h^−1^) and converted with excellent yield (close to 100% of the theoretical maximum) to iBuOH while ethanol was formed in only small amounts from acetate as by-product.

Finally, we could also show that the strain keeps a high specific glucose uptake rate in a pulsed fed-batch bioreactor cultivation, even if cell growth is limited due to accumulation of iBuOH.

## Results

### Stoichiometric metabolic modeling

In order to further improve iBuOH production in *E. coli*, we started with strain RL3000Δferm-pIBA4 [19] because it showed one of the best performances so far. The strain expresses the pathway from pyruvate to iBuOH from a single plasmid, which enables redox-balanced synthesis of iBuOH from glucose while all remaining fermentation pathways are deleted (Δ*ackA* Δ*frdA* Δ*ldhA* Δ*adhE*) (Fig. 1, top). The expression strengths of the involved enzymes were optimized leading to an improved ratio among the enzymes and thus efficient metabolic flux through the biosynthetic pathway [19]. However, even though it was reported that this optimized strain is able to grow anaerobically in defined minimal medium with glucose as sole carbon source, in our hands and with higher initial ODs (initial OD_420_ ~ 0.15–0.25) we were not able to detect significant biomass formation. We tested phosphate buffered minimal medium (adapted from [26], see “Methods” section) as well as MOPS minimal medium purchased from Teknova (as this medium was used in [19]), but in both cases the cells did not grow.

To better understand these experimental observations, we performed basic stoichiometric calculations based on flux balance analysis (FBA). We used the ECC2 network model of *E. coli* [27], extended with the heterologous iBuOH production pathway, and the *CellNetAnalyzer* toolbox [28, 29] to perform these calculations (see “Methods”).

To mimic the experimental setup of the RL3000Δferm-pIBA4 strain under anaerobic conditions, uptake of oxygen as well as the reaction rates of the deleted fermentation pathways (R_ACKr, R_FRD2, R_LDH_D, R_ALCD2x) were set to zero. As expected, with maximization of iBuOH production, FBA showed that all of the taken-up glucose could be converted to iBuOH with a maximal yield of 1 mol_iBuOH_ mol_Glc_^−1^. However, maximization of biomass formation led to a growth rate of zero showing that, according to the model, the strain is indeed not able to grow anaerobically on glucose. Because the pathway from glucose via pyruvate to iBuOH is redox balanced (Fig. 1) and produces ATP, we hypothesized that this infeasibility is due to a redox imbalance induced by the net production of some NADH equivalents when biomass is synthesized from glucose. Similar to our approach employed in [14], we introduced an artificial reaction (R_NADHox) to the network that oxidizes NADH to NAD^+^ (R_NADHox: 1 M_nadh_c + 1 M_h_c → 1 M_nad_c) in order to verify our hypothesis and to calculate the amount of NADH that is causing this imbalance. We then repeated the calculation, this time fixing the growth rate to 0.1 h^−1^ and minimizing R_NADHox. The solution of this optimization problem revealed that 0.015 mmol of NADH are being produced in excess for the synthesis of 0.1 gDW of biomass in this strain. It should be noted that this value depends on the biomass composition used in the model; it increases if the biomass is more oxidized and decreases if it is more reduced.

After identifying the likely cause of impaired cell growth, we aimed to find a practical mechanism that (a) resolves the redox imbalance; (b) does not reduce the high iBuOH/glucose yield; and (3) can be easily applied in *E.coli*. Recently, we solved a similar issue with unbalanced redox equivalents (anaerobic growth of *E. coli* on glycerol in minimal medium [14]) by allowing co-uptake of small amounts of acetate. Acetate was converted via acetyl-CoA to ethanol, consuming 1 mol of ATP per 1 mol of acetate and oxidizing 2 mol of NADH to NAD^+^. This strategy should also be applicable in the case of iBuOH production from glucose but with an altered background strain compared to RL3000Δferm-pIBA4. Since *ackA* and *adhE* are essential for the conversion from acetate to ethanol they need to be kept. Deleting *pflB* instead of *ackA* and *adhE* would block the flux from glucose to formate, ethanol, and acetate but allows the conversion from acetate to ethanol. In this case, acetate would not only act as redox sink in the strain, but would also be needed as an essential precursor for acetyl-CoA synthesis (Fig. 1, bottom). In fact, minimizing the glucose uptake rate for this strain under anaerobic conditions with a fixed growth rate of 0.1 h^−1^ and acetate as co-substrate (with a free flux), we observed that biomass formation is now possible with the following net stoichiometry (H_2_O and H^+^ are not shown and it is assumed that the PDH and FHL are inactive [30, 31]):

1 $$\begin{aligned} & 4.42 {\text{mmol}}_{{{\text{Glc}}}} + 0.44 {\text{mmol}}_{{{\text{Ace}}}} \to \\ & 0.1 {\text{gDW}}_{{{\text{BM}}}} + 3.85 {\text{mmol}}_{{{\text{iBuOH}}}} + 7.90 {\text{mmol}}_{{{\text{CO}}_{2} }} . \end{aligned}$$

Interestingly (and in contrast to anaerobic growth of *E. coli* on glycerol [14]), the model predicts that the uptake of small amounts of acetate without its conversion to ethanol would already suffice to enable redox-balanced growth-coupled iBuOH production. In this case, uptake of acetate is only used to synthesize the biomass precursor acetyl-CoA (or α-ketoglutarate lying downstream of it), which produces one less NADH per acetyl-CoA synthesized compared to generation of acetyl-CoA from glucose. This mechanism is already sufficient in the model to balance the small surplus of NADH generated when building biomass exclusively from glucose (0.015 mmol_NADH_/0.1 gDW_BM_; see above).

### Anaerobic cultivation of strain SB001-pIBA4 and RL3000Δferm-pIBA4 in minimal medium

To verify the hypothesis that uptake of small amounts of acetate should enable cell growth in an iBuOH producer strain under anaerobic conditions, *E. coli* strain SB001 (Δ*pflB* Δ*frdA* Δ*ldhA*) was constructed and transformed with plasmid pIBA4, resulting in strain SB001-pIBA4. Note, that we only focused on interventions in the core carbon metabolism, while other deletions originally introduced in strain RL3000Δferm-pIBA4 (Δ*ilvC* and Δ*lacY*) were not considered in our strain. SB001-pIBA4 and, for comparison, RL3000Δferm-pIBA4 were cultivated anaerobically in minimal glucose medium, both with and without acetate supplementation. As explained above, the model predicted that very low amounts of acetate (around 1/10 of the amount of added glucose) should be sufficient to balance redox. However, to ensure that acetate is not limiting during cultivation, 25 mM of acetate per 15 g L^−1^ (~ 83 mM) glucose was added to the medium, which is somewhat more than the stoichiometric demand calculated. Even though acetate is often considered a growth-inhibiting compound for *E. coli*, it was shown that the growth rate of *E. coli* is only affected to a small extent at this concentration [32, 33]. Moreover, in [32] it was shown that uptake and metabolization of acetate via the Pta-AckA pathway is thermodynamically controlled and requires higher extracellular acetate concentrations.

**Fig. 2:**
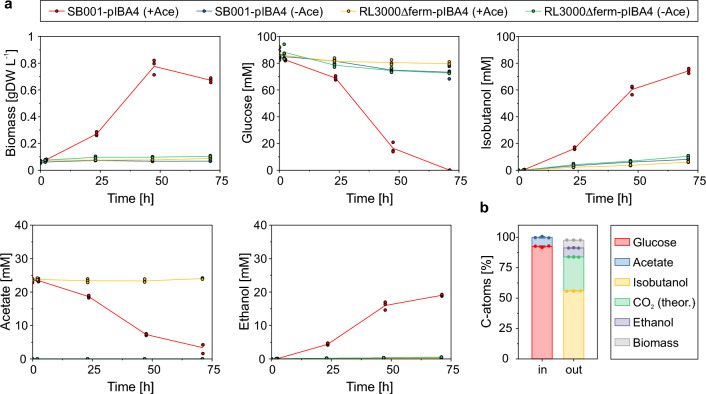
**a** Time courses of biomass, glucose, iBuOH, acetate, and ethanol concentrations of strains SB001-pIBA and RL3000Δferm-pIBA4 cultivated anaerobically in minimal medium with (+ Ace) and without (−Ace) acetate supplementation. The means (solid lines) and individual data (dots) of *n* = 3 biologically independent samples are shown. **b** Carbon balance of biomass and quantified metabolites consumed (in) and produced (out) by strain SB001-pIBA in minimal medium with acetate supplementation. A molar ratio of 2 mol_CO2_ mol_iBuOH_^−1^ were assumed to calculate the amount of produced CO_2_ and a biomass sum formula of C_1.00_H_1.77_O_0.49_N_0.24_ to calculate the amount of C-atoms contained in the produced biomass. The amount of biomass and metabolites were normalized to the number of C-atoms they are composed of. The means (solid columns) and individual data (dots) of *n* = 3 biologically independent samples are shown. The error bars represent s.d

As depicted in Fig. 2a and as predicted by the stoichiometric calculations, strain SB001-pIBA4 was able to grow exponentially (*µ* = 0.051 h^−1^) in minimal medium if acetate was supplemented while no cell growth could be detected if acetate was left out of the medium. RL3000Δferm-pIBA4 grew under none of the two tested cultivation conditions confirming the results obtained before. In principle, both strains took up glucose and produced iBuOH with and without acetate supplementation, but with significantly different rates. In accordance with earlier observations showing that growing cells usually exhibit a higher metabolic activity and thus also a higher specific substrate uptake rate (and specific productivity) than growth-arrested cells [7], the specific glucose uptake rate (4.80 mmol gDW^−1^ h^−1^) and iBuOH productivity (4.36 mmol gDW^−1^ h^−1^) of SB001-pIBA4 with acetate supplementation was between two to three times higher compared to the other strains and cultivation conditions (Table 1). As biomass acts as the catalyst for iBuOH synthesis, the volumetric productivity (calculated for the time interval 2–48 h for each cultivation) of strain SB001-pIBA4 with added acetate (*q*_Iso_ = 1.33 mmol L^−1^ h^−1^) is ten times higher compared to the other cultivations (Table 1), indicating again the advantage of exponentially growing cells compared to resting cells.

**Table 1:** Growth rates, substrate uptake rates, product excretion rates, ATP maintenance rate, product yields, and volumetric iBuOH productivity for strains SB001-pIBA4 and RL3000Δferm-pIBA4 cultivated anaerobically in minimal medium with (+ Ace) and without (−Ace) acetate

	SB001-pIBA4 (+ Ace) *experimental*	SB001-pIBA4 (+ Ace) *FBA*	SB001-pIBA4 (−Ace) *experimental*	RL3000∆ferm-pIBA4 (+ Ace) *experimental*	RL3000∆ferm-pIBA4 (−Ace) *experimental*
*µ* [h^−1^]	0.051 ± 0.001	0.051 (fixed)	~ 0	~ 0	~ 0
*r*_Glc_up_ [mmol gDW^−1^ h^−1^]	4.80 ± 0.06	4.80 (fixed)	2.46 ± 1.33	1.15 ± 0.28	2.42 ± 0.99
*r*_Iso_ex_ [mmol gDW^−1^ h^−1^]	4.36 ± 0.04	4.36 (fixed)	1.60 ± 0.16	1.14 ± 0.02	1.61 ± 0.08
*r*_Ace_up_ [mmol gDW^−1^ h^−1^]	1.18 ± 0.10	1.18 (fixed)	~ 0	~ 0	~ 0
*r*_Eth_ex_ [mmol gDW^−1^ h^−1^]	1.15 ± 0.02	0.93 (calculated)	~ 0	~ 0	~ 0
*r*_CO2_ex_ [mmol gDW^−1^ h^−1^]	n.d	9.77 (calculated)	n.d	n.d	n.d
*r*_ATPM_ [mmol gDW^−1^ h^−1^]	n.d	4.05 (calculated)	n.d	n.d	n.d
*Y*_Iso/Glc_ [mol mol^−1^]	0.89 ± 0.02	0.91 (fixed by given rates)	0.60 ± 0.16	0.67 ± 0.21	0.70 ± 0.38
*Y*_Eth/Ace_ [mol mol^−1^]	0.96 ± 0.05	0.79 (calculated)	n.d	n.d	n.d
*Y*_BM/Glc_ [gDW g^−1^]	0.057 ± 0.001	0.059 (calculated)	~ 0	~ 0	~ 0
*q*_Iso_ (2–48 h) [mmol L^−1^ h^−1^]	1.33 ± 0.03	(n.d. by FBA)	0.14 ± 0.00	0.08 ± 0.00	0.15 ± 0.01

Columns indicated with “experimental” display measured data, while the column indicated with “FBA” displays results from flux balance analysis where the measured rates indicated with “fixed” were used as input for calculating the fluxes indicated as “calculated”. For the calculation of unknown rates, it was assumed that the substrate not used for growth was utilized to synthesize ATP for non-growth-associated maintenance (r_ATPM_). The means ± s.d. of *n* = 3 biologically independent samples are shown. Note that the measured yields may deviate from the respective ratios of measured rates because yields were determined from the final product concentration at the end of the cultivation while rates were calculated for the exponential phase. The volumetric productivity of iBuOH (q_Iso_) was calculated for the time interval 2–48 h for each cultivation

The overall carbon balance of measured metabolites and biomass sums up almost perfectly to 100% (carbon recovery: 97.8%, Fig. 2b). In contrast to the calculated stoichiometry in Eq. (1), the strain excreted ethanol. Hence, a significant part of the acetate taken up is directly used to oxidize NADH to NAD^+^ (by its conversion to ethanol via acetyl-CoA) and the amount of acetate taken up per gram biomass produced is therefore higher than predicted by the model. There are two possible explanations for this observation: the biomass might be more oxidized than the biomass stoichiometry used in the stoichiometric model, leading to a larger excess of NADH than the calculated low value of 0.015 mmol_NADH_/0.1 gDW_BM_. Another explanation would be that a larger fraction of the consumed glucose is oxidized via the oxidative pentose phosphate pathway; the generated NADPH is then converted to NADH (via transhydrogenase) and the latter needs finally to be oxidized in the ethanol pathway.

We used the obtained experimental data for SB001-pIBA4 (+ Ace) as input for flux analyses with the ECC2-iBuOH model (Table 1, column “SB001-pIBA4 (+ Ace) FBA”). Due to redundancies in the rates, fixing all excretion and uptake rates to their measured values gives an infeasible system. Thus, all rates except for the ethanol excretion rate were fixed and the non-growth-associated ATP maintenance rate (*r*_ATPM_) was used as objective function and maximized. Given that the growth rate *µ* was also fixed, pursuing maximization of growth (as typically done in FBA) would not be relevant in this scenario. Consequently, optimizing ATP synthesis appears reasonable in this setting. The FBA predictions led to a slightly smaller ethanol excretion rate compared to the measured values (0.93 mmol gDW^−1^ h^−1^ vs. 1.15 mmol gDW^−1^ h^−1^) and accompanied with the lower rate also to a lower ethanol/acetate yield (0.79 mol mol^−1^ vs. 0.97 mol mol^−1^) (Table 1). The CO_2_ excretion rate was calculated to be 9.77 mmol gDW^−1^ h^−1^ and *r*_ATPM_ was 4.05 mmol gDW^−1^ h^−1^, which is in good accordance with the generally accepted value of 3.15 mmol gDW^−1^ h^−1^ in the literature [34]. Overall, the FBA highlights a good agreement of the measured data with the model predictions.

### Bioreactor cultivation of strain SB001-pIBA4 with gas stripping

Previously, it was reported that high amounts of iBuOH in the medium are toxic for *E. coli* and inhibit growth and viability of the cells [35–37]. To find out, at which iBuOH levels growth of SB001-pIBA4 and iBuOH production are impaired, the cultivations described above were repeated with higher initial glucose and acetate concentrations or with feeding additional glucose and acetate during the cultivation process (data not shown). In all cultivations tested, cell growth stopped when around 70–80 mM (5.2–5.9 g L^−1^) of iBuOH accumulated in the medium. After this threshold, glucose uptake and iBuOH production continued even though the cells did not grow anymore until a final iBuOH titer of around 165 mM (12.2 g L^−1^) was reached. Beyond this iBuOH concentration, glucose uptake stopped and iBuOH production came to a halt. Consequently, higher product titers and a higher volumetric glucose uptake rate can only be reached by in situ removal of iBuOH from the medium. The removal could either be performed with an organic overlay and a liquid–liquid extraction as was shown for a cell-free iBuOH production system [38] or by gas stripping via aeration of the culture broth with nitrogen gas [19, 21, 36]. To investigate whether higher biomass concentrations and higher amounts of iBuOH can be obtained with strain SB001-pIBA4, it was cultivated as pulsed fed-batch fermentation in a bioreactor. The initial glucose and acetate concentrations were doubled compared to the small-scale batch cultivations described above and feeding solution was added in pulses throughout the cultivation before glucose was depleted in the medium (see “Methods” section). Stirrer speed as well as the nitrogen aeration rate was increased during the cultivation to remove as much iBuOH from the medium as allowed by the bioreactor specifications.

**Fig. 3:**
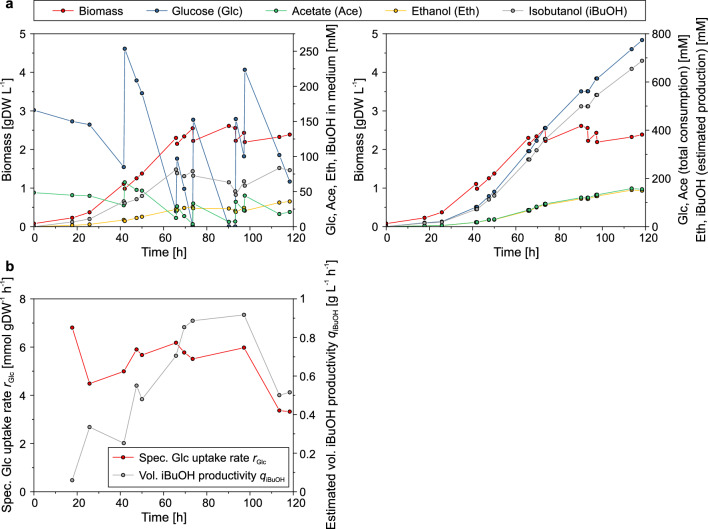
**a** Time courses of biomass, glucose, iBuOH, acetate, and ethanol concentrations in the medium (left) and accumulated acetate and glucose consumption as well as estimated total production of ethanol and iBuOH (right) of strain SB001-pIBA in pulsed fed-batch bioreactor cultivation. The theoretical iBuOH and ethanol product titers were calculated from the total consumption of acetate and glucose with the experimentally determined yields of ethanol/acetate (0.96 mol mol^−1^) and iBuOH/glucose (0.89 mol mol^−1^) (see Table 1). **b** Time courses of specific glucose uptake rate and estimated volumetric iBuOH productivity of strain SB001-pIBA in pulsed fed-batch bioreactor cultivation. A corresponding replicate of the cultivation is shown in Additional file 1: Fig. S1

Even though the medium was sparged continuously with nitrogen gas, iBuOH accumulated in the culture broth (Fig. 3a). Again, the cells grew well reaching a biomass concentration of 2.5 gDW L^−1^ until the iBuOH level in the medium exceeded 80 mM after 66 h. From this time point on, biomass as well as iBuOH concentrations in the medium stayed roughly at a constant level, indicating a balance of iBuOH production and stripping. After diluting the medium with feeding solution (and thus lowering the iBuOH concentration), cells started to grow for a short period, but the biomass concentration oscillated around 2.5 gDW L^−1^ until the fermentation was terminated. Despite growth-arrest, the glucose uptake rate remained at a high level (5.0–6.5 mmol gDW^−1^ h^−1^) throughout the whole cultivation and only decreased to 3.4 mmol gDW^−1^ h^−1^ after ~ 110 h (Fig. 3b). Because we were not able to quantify the iBuOH and ethanol concentration in the off-gas of the bioreactor, the total amount of produced iBuOH and ethanol were estimated from total glucose and acetate consumption using the iBuOH/glucose and ethanol/acetate yields obtained from the small-scale batch cultivations (Fig. 3a, Table 1). Although the calculated rates and amount of produced iBuOH are only estimated values, they demonstrate the great potential of the generated production strain. The (theoretical) volumetric productivity reached peak values beyond 0.9 g_iBuOH_ L^−1^ h^−1^ (≈ 12.4 mmol L^−1^ h^−1^) after 100 h of cultivation (Fig. 3b) and overall 688 mmol of iBuOH were produced per liter after 118 h of cultivation (Fig. 3a). With these values, our approach outperforms reported bioreactor cultivations with strains that need yeast extract addition for biomass formation (like RL3000Δferm-pIBA4), where an iBuOH titer of ~ 500 mM after ~ 195 h was reached. [19]. The described bioreactor run was repeated with a higher initial biomass concentration (OD_420_ of 0.84; Additional file 1: Fig. S1). The obtained theoretical iBuOH titer after 125 h of cultivation (584 mM) as well as the specific glucose uptake rate (2.4–5.1 mmol gDW^−1^ h^−1^) and (theoretical) iBuOH peak productivity (0.7 g_iBuOH_ L^−1^ h^−1^) were slightly reduced but still comparable with the values obtained with the first bioreactor run. Interestingly, the steady-state iBuOH concentration in the medium was lower (43 mM vs. 81 mM; possibly due to a more effective isobutanol stripping), allowing the cells to grow to a higher density of 3.75 gDW L^−1^.

## Discussion

Since the first successful heterologous production of iBuOH in *E. coli* [16], optimizing the production strain and cultivation conditions was focus of research over the last 15 years. One central part of these optimization steps was the modulation of cofactors and redox equivalents required for anaerobic iBuOH synthesis [17, 18, 39–41]. However, in 2011 it was already shown in a computational study that homofermentative synthesis of iBuOH as sole product accompanied with biomass formation is not feasible in defined minimal medium due to redox imbalances [24, 25]. Thus, all *E. coli* iBuOH producer strains designed so far that are able to form biomass either relied on oxygen [16, 17, 21, 41], the secretion of alternative fermentation products (e.g., ethanol or succinate) [24, 25, 40, 42], and/or supplementation with complex additives such as yeast extract [16–19, 35, 36, 39, 40, 43, 44] to achieve redox balance. How complex additives can enable the cell to regain redox balance was recently shown in [14]. Cultivating *E. coli* under aerobic conditions or enabling the secretion of alternative fermentation products always leads to the disadvantage of a reduced product yield (because carbon from glucose is either lost to CO_2_ via respiration or to other products than iBuOH), whereas the addition of yeast extract increases the overall costs of the production process and leads to varying growth behavior due to differences in the composition of this complex supplement [21, 23]. This issue could be addressed by cultivating the producer strain under *micro*aerobic conditions, providing only very little oxygen to recycle the NADH, which is formed in excess during biomass synthesis (see “Results” section), via respiration. However, microaerobic conditions are hard to control especially in larger scale bioreactor cultivations making this strategy not feasible for industrial applications. Alternatively and as already tested for iBuOH production in *E. coli*, a two-stage process could be applied [41]. Here, the cells are cultivated under (fully) aerobic conditions for biomass accumulation in the first part of the process and then shifted to (fully) anaerobic conditions for iBuOH synthesis without growth in the second part. Using this process, the overall yield should be similar to a microaerobic process if initial substrate concentration and final biomass concentration are the same in both processes. However, under growth-arrest, *E. coli* tends to slow-down its metabolism and thus specific glucose uptake rate and iBuOH production rate decrease, leading to an overall lower volumetric productivity ([3, 7, 8, 11], Table 1).

The iBuOH production process with acetate as co-substrate suggested in this study addresses most of the issues mentioned above. Although strictly speaking it is not a homofermentative process (as ethanol is secreted in small amounts as a by-product), iBuOH is the only fermentation product that is directly formed from the main substrate glucose with a yield close to the theoretical maximum (89% of the maximal yield of 1 mol mol^−1^). This high iBuOH/glucose yield is due to the deletion of the *pflB* gene that blocks the flux from glucose to the alternative fermentation products formate, acetate, and ethanol while the synthesis of ethanol from acetate is still possible. The next-generation feedstock “green acetate” can be sustainably generated from lignocellulosic biomass in biorefineries or from C1 gases through acetogenic organisms [45, 46]. Since acetate and its subsequent conversion to ethanol acts as the needed redox sink for biomass formation in strain SB001-pIBA4, oxygen or yeast extract are not essential for redox balancing. Thus, this process can be conducted under strict anaerobic conditions, which is the preferred *modus operandi* for industrial large-scale cultivations and the cells are able to grow exponentially (and reproducibly), not impacted by varying compositions of expensive and complex supplements such as yeast extract or peptone and the calculated yields are not distorted by uptake of these supplements.

Due to the enabled growth, the metabolic activity and thus specific glucose uptake rate (and iBuOH productivity) are considerably higher in SB001-pIBA4 compared to the cultivations with growth-arrested cells (Table 1). Consequently, the strain is not only suitable for one-stage anaerobic fermentations, but could also be employed in a two-stage cultivation strategy. Here, the strain would first be cultivated aerobically (without induction of the iBuOH pathway) to accumulate biomass and then shifted with induced iBuOH pathway to anaerobic conditions in the second stage. Ideally, acetate, produced as an overflow metabolite in the aerobic phase [47], could then directly be recycled for (slow) cell growth in the anaerobic production phase, leading to an overall higher carbon utilization and volumetric productivity. Recently, it was shown in a computational study that slow growth in stage two is favorable over a production-only stage with growth arrest regarding an optimal TRY distribution [4]. Following this two-stage approach, one would need to calculate the optimal time point for switching from aerobic to anaerobic growth for obtaining the best (volumetric) productivity (under side constraints of a demanded minimum yield).

To exploit the full potential of strain SB001-pIBA4 (for one- or two-stage processes), a more efficient way for in situ removal of iBuOH needs to be found. Despite rigorous sparging with nitrogen, iBuOH accumulated at concentrations high enough for growth inhibition after 66 h of cultivation and a biomass concentration of ~ 2.5 gDW L^−1^ in the bioreactor (Fig. 3). Thus, volumetric productivity could not increase further from that time point on. Different methods for removal of iBuOH from aqueous solutions have been investigated [36, 38, 48–50], but the ones relying on liquid–liquid extraction with non-biocompatible solvents are not directly applicable to iBuOH removal during the course of fermentation. A perfusion process with external liquid–liquid extraction or other strategies established for a similar issue during ABE fermentation [51, 52] could be suitable to address the in situ product recovery and thus productivity of strain SB001-pIBA4.

Together with few other studies [14, 53, 54], this work demonstrates the beneficial use of acetate as redox sink to enable growth and production under strict anaerobic conditions. We believe that there are other potential applications, with different hosts or/and substrate–product combinations, where this approach could be applied. For example, 2,3-butanediol (2,3-BDO) production in *E. coli* is only possible under microaerobic conditions in minimal glucose medium [10, 55] because the pathway is not redox balanced (2 molecules of NADH are produced during glycolysis but only 1 molecule is consumed from pyruvate to 2,3-BDO). Using SB001 as background strain and providing acetate in the medium should also enable 2,3-BDO production and biomass formation under strict anaerobic conditions. However, as not only NADH from biomass synthesis, but also one additional NADH for every formed 2,3-BDO molecule needs to be recycled, acetate uptake (and ethanol secretion) will be considerably higher compared to iBuOH production (0.5 molecules of acetate per molecule of formed 2,3-BDO would additionally be needed). Furthermore, as the uptake of one molecule of acetate and its conversion to ethanol costs one molecule of ATP, the ATP yield would be lower.

## Methods

### Strains, media, and culture conditions used in this study

All strains, plasmids, and primers used in this study are listed in Table 2. The *frdA* gene was deleted in strain LK3S by homologous recombination using a deletion cassette generated by PCR from plasmid pKD4 and primer pair P1_frdA_fw/P2_frdA_rv [56]. The introduced kanamycin resistance cassette was afterwards excised by the FLP-recombinase [56], leading to strain SB001.

**Table 2:** Strains, plasmids, and primers used in this study

Strain or plasmid	Description	Reference
RL3000Δferm	*E. coli* MG1655 *ilvG*^+^ *rph*^+^ *rfb*-50 *ybhJ*(L54→I) *yebN*(G25→D) *ycfK*::97 bp. Δ*insB-5* Δ*insA-5* Δ*insAB-5* Δ*ackA* Δ*frdA* Δ*ldhA* Δ*adhE* Δ*ilvC* Δ*lacY*	[19]
LJ110	*E. coli* W3110 Fnr^+^	[57]
LK3S	LJ110 Δ*ldhA* Δ*pflB*	Lab collection
SB001	LJ110 Δ*ldhA* Δ*pflB* Δ*frdA*	This study
RL3000Δferm-pIBA4	RL3000Δferm transformed with plasmid pIBA4	[19]
SB001-pIBA4	SB001 transformed with plasmid pIBA4	This study
pIBA4	Expression plasmid encoding the iBuOH pathway [pBBRori-*aadA1*(Sp^R^)-T_L_-*lacI*-P_*lacIq*_] (814bb)-P_T7A1-O34_-RBS_AS7_-*alsS*_*Bsu*_-RBS_AC7_-*ilvC*_*Eco-*6E6_-RBS_AD5_-*ilvD*_*Eco*_-RBS_AK7_-*kivd*_*Lla*_-RBS_AA2_-*adhA*_*Lla-*M5_-*gfp*-T_P22_	[19]
pKD4	Template plasmid for generation of deletion cassette	[56]
**Primer name**	**Sequence (5ʹ-3ʹ)**	
P1_frdA_fw	CAAACCTTTCAAGCCGATCTTGCCATTGTAGGCGCCGTGTAGGCTGGAGCTGCTTCG	
P2_frdA_rv	CGCCTTCTCCTTCTTATTGGCTGCTTCCGCCTTATCCATATGAATATCCTCCTTA	

All liquid and solid media used for cultivating strains RL3000Δferm-pIBA4 and SB001-pIBA4 contained 50 µg mL^−1^ spectinomycin. For growth assays under anaerobic conditions, a single colony of the respective strain was picked from an LB_0_ agar plate (10 g L^−1^ tryptone, 5 g L^−1^ yeast extract, 5 g L^−1^ NaCl, 15 g L^−1^ agar) and transferred to 5 mL of LB_0_ liquid medium (10 g L^−1^ tryptone, 5 g L^−1^ yeast extract, 5 g L^−1^ NaCl,) and incubated at 37 °C and 200 rpm for 5 h. From the LB_0_ culture, 100 mL of chemically defined minimal medium (MM: 15 g L^−1^ glucose, 34 mM NaH_2_PO_4_, 64 mM K_2_HPO_4_, 20 mM (NH_4_)_2_SO_4_, 1 μM Fe(SO_4_)_4_, 300 μM MgSO_4_, 1 μM ZnCl_2_, 10 μM CaCl_2_, 250 µM IPTG, adapted from [26]) were inoculated with a 1:50 dilution. (Note that, despite the addition of IPTG, the notion of minimal medium is used herein to indicate the absence of undefined carbon sources such as yeast extract). When indicated, the medium was supplemented with 25 mM of sodium acetate. The MM cultures were incubated without shaking in a Whitley A25 anaerobic workstation (Don Whitley Scientific Limited) with an oxygen-free atmosphere (80% N_2_, 10% CO_2_ 10% H_2_) at 30 °C for 20 h. For the main culture, the cells were washed once in MM and transferred to 25 mL of fresh MM (in a 25-mL Schott bottle) with an optical density at 420 nm (OD_420_) of 0.25 and incubated in the anaerobic workstation at 30 °C, stirred with a magnetic stirrer at 200 rpm.

The pulsed fed-batch bioreactor cultivation of strain SB001-pIBA4 was carried out in a Multifors bioreactor (Infors AG) with a starting volume of 400 mL and an initial OD_420_ of 0.35 (OD_420_ of 0.84 for the replicate, see Additional file 1: Fig. S1). The pre-culture was prepared as described above, but 200 mL of MM was used instead of 100 mL and the MM pre-culture was incubated in the anaerobic workstation for 48 h. The cultivation was performed in MM with increased initial glucose and sodium acetate concentrations (30 g L^−1^ and 50 mM, respectively). The temperature was set to 30 °C and the pH maintained at 6.9 by the addition of 1 M HCl or 1 M NaOH. During the first 18 h of cultivation, the stirrer speed was set to 300 rpm and to guarantee anaerobic conditions, the bioreactor was aerated with N_2_ with a rate of 0.8 L min^−1^ via headspace. After 18 h, the stirrer speed was increased to 750 rpm and the N_2_-aeration switched to sparger aeration to remove iBuOH from the medium. During the cultivation, the stirrer speed and aeration rate were further increased to strip as much iBuOH from the medium as possible (after 42 h: 800 rpm and 1.35 L min^−1^; after 47.5 h: 875 rpm and 1.35 L min^−1^; after 66.5 h: 950 rpm and 1.5 L min^−1^). Feeding solution (240 g L^−1^ glucose, 300 mM sodium acetate, 34 mM NaH_2_PO_4_, 64 mM K_2_HPO_4_, 20 mM (NH_4_)_2_SO_4_, 1 μM Fe(SO_4_)_4_, 300 μM MgSO_4_, 1 μM ZnCl_2_, 10 μM CaCl_2_, 250 µM IPTG, 50 µg mL^−1^ spectinomycin) were added at following volumes and time points: after 42 h → 50 mL feeding solution; after 66.25 h → 25 mL feeding solution; after 73.75 h → 50 mL feeding solution; after 93.42 h → 50 mL feeding solution; after 97.58 h → 50 mL feeding solution (for the replicate: after 28.25 h → 50 mL feeding solution; after 52.25 h → 33.3 mL feeding solution; after 77.25 h → 50 mL feeding solution; after 101 h → 50 mL feeding solution, see Additional file 1: Fig. S1). The time points and the volumes of feeding solution additions were estimated from the previously determined glucose uptake rate such that the feeding solution was added before glucose was depleted in the medium and that the glucose level in the medium stayed below ~ 300 mM.

For all cultivations, cell growth was monitored by measuring the OD_420_ and using a conversion factor of 0.22 gDW OD_420_^−1^ to obtain the concentration of dry biomass in gDW L^−1^ (gDW: gram dry weight).

### Quantification of metabolites

Glucose and ethanol were quantified using the Megazyme D-Glucose Assay Kit (K-GLUC) and the Megazyme Ethanol Assay Kit (K-ETOH) according to the manufacturer’s instructions. Acetate was quantified either by the Megazymes Acetic Assay Kit (K-ACET) or by HPLC as described previously [9]. Formate, pyruvate, lactate, succinate, fumarate, and orotate were measured by the same HPLC method but could not be detected in the medium. iBuOH quantification was performed by GC–MS/MS in SIM mode using an Agilent 5975C inert XL EI/CI MSD system (Agilent Technologies) upgraded to MS/MS with an Evolution3 system (Chromtech) and equipped with an HP-5MS Ultra Inert column (dimensions: 30 m, 0.25 mm, 0.25 µm, Agilent Technologies) and a Combi PAL-XT auto sampler (CTC Analytics). 1 mL of saturated NaCl solution was mixed with 100 µL of sample in a headspace GC vial and sealed. The vial was incubated at 80 °C for 3 min and 500 µL of the head space gas were injected with a 30:1 split at 33.9 mL min^−1^ to the GC–MS/MS system using a 2.5 mL syringe heated to 85 °C. The inlet temperature was set to 250 °C and a constant flow of 1 mL min^−1^ helium was used as carrier gas. The oven temperature was held at 33 °C for 2 min followed by a linear temperature gradient of 25 °C min^−1^ to a final temperature of 200 °C. Mass spectra were recorded starting 1.48 min after injection and *m/z* values of 55.0, 56.0, and 74.0 were used to detect iBuOH in SIM mode at 6.67 scans s^−1^. Chromatograms were evaluated with the Agilent ChemStation software (Agilent Technologies) or the GNPS dashboard [58]. For quantification of iBuOH, a calibration curve was prepared with external standards based on their determined peak areas.

### Determination of growth rates, excretion and uptake rates, productivity, and yield

The measured biomass and metabolites in the medium were used to calculate the corresponding growth rate *µ* and excretion/uptake rates *r*_M_, or productivity *q*_M_, respectively. All rates and yields were first determined for each replicate and the means and standard deviations were calculated with the Origin Pro 2020b software (OriginLab Corp.) afterwards.

For exponentially growing cells, *µ* was determined by plotting the natural logarithm of the biomass concentrations of each sampled time point (within the exponential growth phase) against the cultivation time. The slope of the linear regression equals *µ*. Excretion and uptake rates were calculated with the formula:

$$r_{{\text{M}}} = \mu \cdot \frac{{c_{{{\text{M}},{\text{e}}}} - c_{{{\text{M}},{\text{s}}}} }}{{X_{{\text{e}}} - X_{{\text{s}}} }} \left[ {{\text{mmol }} \cdot {\text{gDW}}^{{ - 1}} \cdot {\text{h}}^{{ - 1}} } \right],$$

where *µ* is the growth rate (in h^−1^), *c*_M,e_ and *c*_M,s_ represent the end and start concentration of the respective metabolite M (in mM), and *X*_e_ and *X*_s_ represent the end and start concentration of biomass (in gDW L^−1^).

For non-growing cells, excretion and uptake rates were calculated with the formula:

$$r_{{\text{M}}} = \frac{{c_{{{\text{M}},{\text{e}}}} - c_{{{\text{M}},{\text{s}}}} }}{{X_{{{\text{Av}}}} \cdot \Delta t}} \left[ {{\text{mmol }} \cdot {\text{gDW}}^{{ - 1}} \cdot {\text{h}}^{{ - 1}} } \right],$$

where *X*_Av_ is the average biomass concentration (in gDW L^−1^) within the respected time period, and Δ*t* (= *t*_e_ − *t*_s_) the length of the time period (in h).

Volumetric productivities *q*_M_ were calculated with the formula:

$$q_{{\text{M}}} = \frac{{c_{{{\text{M}},{\text{e}}}} - c_{{{\text{M}},{\text{s}}}} }}{\Delta t} \left[ {{\text{mmol }} \cdot {\text{L}}^{{ - 1}} \cdot {\text{h}}^{{ - 1}} } \right].$$

Product yields *Y*_P/S_ were determined by plotting Δc_P_ (in mM) against Δc_S_ (in mM) for every sampled time point. The slope of the linear regression equals *Y*_P/S_ (in mol mol^−1^). The biomass yield *Y*_BM/Glc_ was determined by plotting Δc_BM_ (in gDW L^−1^) against Δc_Glc_ (in g L^−1^) for every sampled time point in the exponential growth phase. The slope of the linear regression equals *Y*_BM/Glc_ (in gDW g^−1^).

### Stoichiometric calculations and FBA

For all stoichiometric calculations and FBA we used the *EColiCore2* (ECC2) network model [27] and the *CellNetAnalyzer* toolbox [28, 29]. The ECC2 model was extended to allow heterologous iBuOH production by adding iBuOH as (external) metabolite (M_iBuOH_e) and the following net reaction equation for iBuOH synthesis from pyruvate (the involved enzymes are encoded on plasmid pIBA4): R_iBuOH: 4 M_h_c + 2 M_nadh_c + 2 M_pyr_c → 2 M_co2_c + 1 M_h2o_c + 2 M_nad_c + 1 M_iBuOH_e. Furthermore, R_PDH was set to zero, as it is not active under anaerobic conditions and R_FHL was also assumed to be inactive.

FBA was used with different objective functions and different deleted reactions (R_ACKr = R_FRD2 = R_LDH_D = R_ALCD2x = 0 for background strain RL3000Δferm; R_FRD2 = R_LDH_D = R_PFL = 0 for background strain SB001) to analyze anaerobic metabolism and iBuOH production of each strain in minimal medium (for details see “Results” section).

### Supplementary Information

**Additional file 1: Figure S1.** Time courses of biomass, glucose, iBuOH, acetate, and ethanol concentrations in the medium and accumulated acetate and glucose consumption as well as estimated total production of ethanol and iBuOH of strain SB001-pIBA in a second pulsed fed-batch bioreactor cultivation.

## Data Availability

All data generated or analyzed during this study are included in this published article.
