# Supplementary material for: Growth-coupled anaerobic production of isobutanol from glucose in minimal medium with Escherichia coli
Source: Biotechnol Biofuels Bioprod. 2023 Oct 3;16:148. doi: 10.1186/s13068-023-02395-z (PMC10548627; doi:10.1186/s13068-023-02395-z)
Supplement: Supplementary file 1 — Additional file 1: Figure S1. Time courses of biomass, glucose, iBuOH, acetate, and ethanol concentrations in the medium and accumulated acetate and glucose consumption as well as estimated total production of ethanol and iBuOH of strain SB001-pIBA in a second pulsed fed-batch bioreactor cultivation. [file 13068_2023_2395_MOESM1_ESM.docx]

**Additional File 1**

**Growth-coupled anaerobic production of isobutanol from glucose in minimal medium with *Escherichia coli***

Simon Boecker^1^, Peter Schulze^2^, Steffen Klamt^1,*^

^1^Analysis and Redesign of Biological Networks, Max Planck Institute for Dynamics of Complex Technical Systems, Sandtorstr. 1, 39106 Magdeburg, Germany

^2^Physical and Chemical Foundations of Process Engineering, Max Planck Institute for Dynamics of Complex Technical Systems, Sandtorstr. 1, 39106 Magdeburg, Germany

^*^Corresponding author: [klamt@mpi-magdeburg.mpg.de](mailto:klamt@mpi-magdeburg.mpg.de)

**
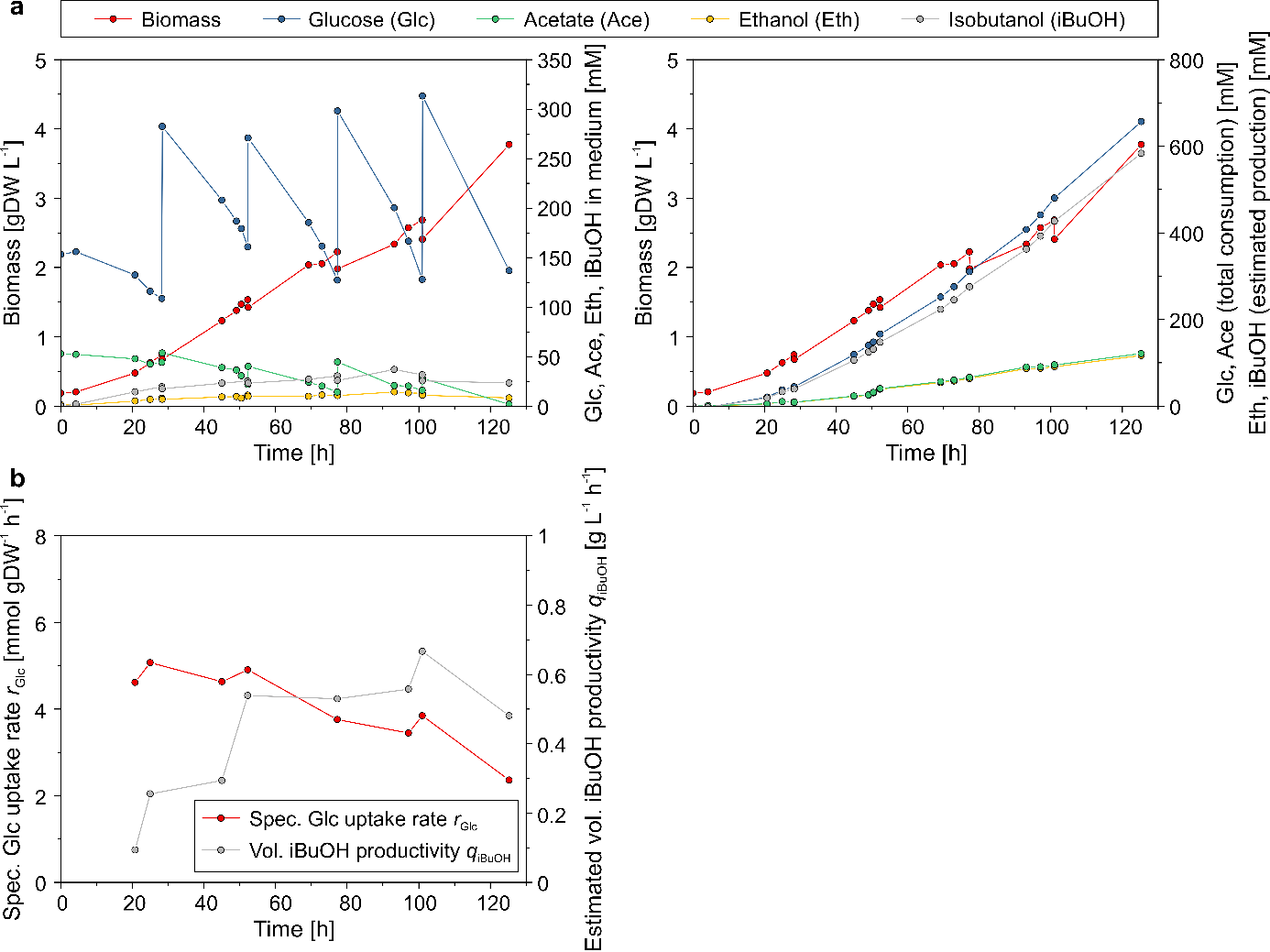
**

**Figure S1: a) Time courses of biomass, glucose, iBuOH, acetate, and ethanol concentrations in the medium (left) and accumulated acetate and glucose consumption as well as estimated total production of ethanol and iBuOH (right) of strain SB001-pIBA in a second pulsed fed-batch bioreactor cultivation.** The theoretical iBuOH and ethanol product titers were calculated from the total consumption of acetate and glucose with the experimentally determined yields of ethanol/acetate (0.96 mol mol^-1^) and iBuOH/glucose (0.89 mol mol^-1^) (see Table 1). **b)** **Time courses of specific glucose uptake rate and estimated volumetric iBuOH productivity of strain SB001-pIBA in pulsed fed-batch bioreactor cultivation.** A corresponding replicate of the cultivation is shown in Figure 3.
